# Carbapenem-resistant hypermucoviscous *Klebsiella pneumoniae* clinical isolates from a tertiary hospital in China: Antimicrobial susceptibility, resistance phenotype, epidemiological characteristics, microbial virulence, and risk factors

**DOI:** 10.3389/fcimb.2022.1083009

**Published:** 2022-12-21

**Authors:** Qiang Wang, Mengyuan Chen, Qian Ou, Lina Zheng, Xuejing Chen, Guofeng Mao, Jiaqi Fang, Dazhi Jin, Xiaofang Tang

**Affiliations:** ^1^ Department of Clinical Laboratory, the Second Affiliated Hospital of Zhejiang Chinese Medical University, Hangzhou, Zhejiang, China; ^2^ Department of Basic Medical Sciences, Zhejiang University School of Medicine, Hangzhou, China; ^3^ Department of Clinical Laboratory, Shaoxing People’s Hospital, Shaoxing, China; ^4^ Department of Clinical Medicine, Zhejiang University City College, School of Medicine, Hangzhou, China; ^5^ School of Laboratory Medicine, Hangzhou Medical College, Hangzhou, China; ^6^ Key Laboratory of Biomarkers and In Vitro Diagnosis Translation of Zhejiang Province, Hangzhou, China; ^7^ Department of Cadre Health Care, Zhejiang Hospital, Hangzhou, China

**Keywords:** *Klebsiella pneumoniae*, carbapenemase, antibiotic resistance, hypermucoviscous, KPC-2, OXA-48

## Abstract

Hypervirulent and multidrug-resistant *Klebsiella pneumoniae* poses a significant threat to public health. We aimed to determine the common carbapenemase genotypes and the carriage patterns, main antibiotic resistance mechanisms, and *in vitro* susceptibility of clinical isolates of carbapenem-resistant *K. pneumoniae* (CRKP) to ceftazidime/avibactam (CZA) for the reasonable selection of antimicrobial agents and determine whether hypermucoviscous (HMV) phenotype and virulence-associated genes are key factors for CRKP colonization and persistence. Antibiotics susceptibility of clinical CRKP isolates and carbapenemase types were detected. CRKP isolates were identified as hypermucoviscous *K. pneumoniae* (HMKP) using the string test, and detection of virulence gene was performed using capsular serotyping. The *bla*_KPC-2_, *bla*_NDM_, *bla*_IMP_, and/or *bla*_OXA-48-like_ were detected in 96.4% (402/417) of the isolates, and the *bla*_KPC-2_ (64.7%, 260/402) was significantly higher (*P*<0.05) than those of *bla*_NDM_ (25.1%), *bla*_OXA-48-like_ (10.4%), and *bla*_IMP_ (4.2%). Carriage of a single carbapenemase gene was observed in 96.3% of the isolates, making it the dominant antibiotic resistance genotype carriage pattern (*P* < 0.05). Approximately 3.7% of the isolates carried two or more carbapenemase genotypes, with *bla*_KPC-2_ + *bla*_NDM_ and *bla*_NDM_ + *bla*_IMP_ being the dominant multiple antibiotic resistance genotype. In addition, 43 CRKP isolates were identified as HMKP, with a prevalence of 10.3% and 2.7% among CRKP and all *K. pneumoniae* isolates, respectively. Most clinical CRKP isolates were isolated from elderly patients, and carbapenemase production was the main mechanism of drug resistance. Tigecycline and polymyxin B exhibited exceptional antimicrobial activity against CRKP isolates *in vitro*. Furthermore, *bla*_KPC-2_, *bla*_NDM_, and *bla*_OXA-48-like_ were the main carbapenemase genes carried by the CRKP isolates. CZA demonstrated excellent antimicrobial activity against isolates carrying the single *bla*_KPC-2_ or *bla*_OXA-48-like_ genotype. Capsular serotype K2 was the main capsular serotype of the carbapenem-resistant HMKP isolates. Survival rates of *Galleria mellonella* injected with *K. pneumoniae* 1–7 were 20.0, 16.7, 6.7, 23.3, 16.7, 3.3, and 13.3, respectively. Therefore, worldwide surveillance of these novel CRKP isolates and carbapenem-resistant HMKP isolates as well as the implementation of stricter control measures are needed to prevent further dissemination in hospital settings.

## 1 Introduction

*Klebsiella pneumoniae* (KP) is a Gram-negative bacterium belonging to the family Enterobacterales and is characterized by its distinct capsule and expression of fimbriae in most strains. Being a member of the normal intestinal and upper respiratory tract flora in humans and animals, KP often causes hospital-acquired infections, such as bacteremia, respiratory tract infection, enteritis, meningitis, wound infection, and urinary tract infection (Wyres et al., 2020). Currently, carbapenem antibiotics that are widely used for treatment in clinical practices in China include imipenem (IPM), meropenem (MEM), and ertapenem (ETM). According to nationwide bacterial resistance surveillance data in China, the resistance rates of KP to MEM and IPM were 2.9% and 3.0% in 2005, respectively, but increased to 26.3% and 25.0% in 2020, respectively. With the continuous increase in drug resistance among pathogens, antibiotic resistance in carbapenem-resistant KP (CRKP) has progressively worsened as resistance rates show certain regional differences (Hu et al., 2020).

Ceftazidime/avibactam (CZA) is a novel cephalosporin/β-lactamase inhibitor that effectively inhibits type A (primarily KPC), type C, and certain type D (OXA-48) carbapenemases, but is ineffective against type B carbapenemases (Falcone et al., 2021; Karaiskos et al., 2021). Currently, antibacterial drug options for the clinical treatment of CRKP are extremely limited, and the development of polymyxin B and tigecycline resistance in patients has been frequently reported (Jin et al., 2021). CZA, a novel antimicrobial agent, possesses good therapeutic effects and safety (Falcone et al., 2021). It also offers other advantages such as high antibacterial activity against multidrug-resistant gram-negative (GN) bacteria, high patient compliance, few adverse effects, and good clinical efficacy. Therefore, CZA is currently widely used in clinical practice as salvage therapy for treatment in patients infected with CRKP (Oueslati et al., 2019).

A new phenotype, identified as hypermucoviscous (hypervirulent) KP (HMKP), was first described in Taiwan in the 1990s and 1980s and mainly caused serious infections in immunocompetent, young, and healthy individuals (Liu et al., 1986; Wang et al., 1998). Of a more serious note, because of the dissemination of mobile genetic elements encoding carbapenemases, carbapenem-resistant HMKP isolates have been increasingly reported (Martin et al., 2017; Peng et al., 2022). The emergence of carbapenem-resistant HMKP isolates is of great concern as they could cause severe untreatable infections in individuals (Di Domenico et al., 2020). Moreover, capsular polysaccharides (CPS) and virulence-associated genes are remarkable virulence factors of KP that allow this bacterium to pose an important threat to public health (Liu et al., 2014). Among the over 78 CPS types of KP, K1 and K2 (and to a lesser extent K5, K20, K54, and K57) strains overproducing capsular polysaccharides are the most virulent human pathogens (Wang et al., 1998; Liu et al., 2014). Of these virulence factors, CPS are considered the major determinant of KP pathogenesis. As determined by multivariate analysis, strains carrying *rmpA* are significantly associated with the HMV-phenotype (Lin et al., 2004; Yu et al., 2006). Meanwhile, *wcaG* encodes capsular fucose, which may enhance the ability of the bacteria to evade phagocytosis by macrophages (Fu et al., 2018). The siderophore-associated gene *iut*A mainly mediates iron transport in the GN bacteria, which are widely spread among HMKP isolates (Compain et al., 2014). At present, many studies have reported HMKP infections, especially pyogenic liver abscesses. The present study aimed to systematically describe the molecular characteristics of carbapenemases and hypermucoviscous traits, as well as clinical features of CRKP isolates. The present study aimed to provide evidence for the investigation and prevention of CRKP infections worldwide. Moreover, our results indicate that available surveillance and strict infection administration strategies should be implemented to prevent carbapenem-resistant HMKP outbreaks in Hangzhou, eastern China.

## 2 Methods

### 2.1 Sources and identification of clinical isolates

KP isolates that showed non-susceptibility to at least one of the three tested carbapenem antimicrobials (IMP 10 μg, MEM 10 μg, ETM 10 μg) were considered carbapenem-resistant. In total, 417 non-duplicate CRKP isolates (26.5%) were collected from patients hospitalized from a total of 1573 KP isolates at the Zhejiang Chinese Medical University Hospital in Hangzhou, eastern China between January 2019 and December 2020. Among the CRKP strains, 231 were isolated from sputum specimens of patients with lower respiratory tract infection, 98 from urine specimens of patients with urinary tract infection; 16 from sterile fluids, including articular fluid, hydrothorax, and ascitic fluid specimens of patients with infectious diseases; 25 from specimens of patients with wound infection, 27 from the peripheral blood specimens of sepsis patients, 9 strains from bile specimens of patients with biliary tract infection, and 11 strains from arteriovenous catheter of patients with catheter-related infection. The strains were preliminarily identified using the matrix-assisted laser desorption/ionization time-of-flight mass spectrometry (MALDI-TOF MS; VITEK MS, bioMérieux) and were further confirmed using 16S rRNA sequencing. This study was approved by the Ethics Committee of the Second Affiliated Hospital of Zhejiang Chinese Medical University (approval no.: 2022-LW-006-A01).

### 2.2 Antibiotic susceptibility testing

Susceptibilities of 18 antibiotics were determined using the broth microdilution method. The results were interpreted according to the Clinical and Laboratory Standard Institute (CLSI) guidelines to determine the minimum inhibitory concentrations (MICs) of 16 antibiotics against the CRKP isolates, except for tigecycline and colistin (CLSI, 2021). MIC breakpoints of tigecycline for the CRKP isolates (susceptible, ≤2 mg/L; resistant, ≥8 mg/L) were issued by the Food and Drug Administration. Susceptibility of the CRKP isolates to polymyxin B was extrapolated from the European Committee on Antimicrobial Susceptibility Testing (EUCAST) colistin breakpoints (susceptible, ≤2 mg/L; resistant, >2 mg/L) (EUCAST, 2021). The strains used for quality control were *Escherichia coli* ATCC25922, *Pseudomonas aeruginosae* ATCC27853, and *K. pneumoniae* ATCC700603 (National Institute for the Control of Pharmaceutical and Biological Products, Beijing, China).

### 2.3 Phenotypic detection of carbapenemase-producing strains

In accordance with CLSI M100-S31 (CLSI, 2021), combination of the modified carbapenem inactivation method (mCIM) and the EDTA-CIM method (eCIM) was used to detect carbapenemases as described previously (Ramadan et al., 2022): a loopful (1 μL) of *K. pneumoniae* isolates from an overnight blood agar plate was resuspended in a 2-mL tube of tryptic soy broth (TSB) (Oxoid, UK) and in another 2-mL tube of TSB supplemented with EDTA (Solarbio, Beijing, China) at a final concentration of 5 mM (20 μL of 0.5M EDTA was added to 2 mL TSB). A MEM disk was placed in each tube, and the tubes were incubated at 35°C for 4 h. MEM disks were removed and applied to Mueller Hinton (MH) agar plates (Autobio, Zhengzhou, China) freshly plated with a 0.5-McFarland suspension of a carbapenem-susceptible *E. coli* ATCC25922 strain. Plates were incubated at 35°C for 18–24 h. The diameter of the inhibition zone around each MEM disk was then measured.

Results were interpreted as follows: mCIM-positive: carbapenemase-producing bacterium; mCIM-negative: non-carbapenemase-producing bacterium; mCIM-positive and eCIM-negative: serine carbapenemase-producing bacterium; and mCIM-positive and eCIM-positive: metallo-β-carbapenemase (MBL)-producing bacterium. KP ATCC BAA-1706 and KP ATCC BAA-1705 were used as the negative and positive control strains for the mCIM testing, respectively, and KP ATCC BAA-2146 was used as the positive control strain for eCIM testing.

### 2.4 Molecular detection of carbapenemase genes in CRKP isolates

Genomic DNA of the CRKP isolates was extracted using a bacterial genomic DNA extraction kit (GENEray) according to the manufacturer’s instructions and were subjected to measurements for concentration and purity through ultraviolet spectrophotometry. The procedure after bacteria lysis could be accomplished in 20 minutes; the whole procedure included bacteria lysis, DNA binding with column, and DNA purification. The detailed protocol included the standard steps. Primers were synthesized by Invitrogen (Shanghai, China), including those for *bla*_KPC_, *bla*_NDM_, *bla*_IMP_, and *bla*_OXA-48-like_, described previously for CRKP, as shown in **Supplementary Data** (Poirel et al., 2011). A high-fidelity polymerase chain reaction (PCR) kit (Takara Bio) was used to detect the target genes in the isolates. The total volume of the reaction system was 100 μL, consisting of 2.5 mol/L dNTP, 200 nmol/L each of the forward and reverse primers, 20 mol/L MgCl_2_, 2.5 U EX-Taq DNA polymerase, 100 ng DNA template, and 1× PCR buffer (pH 8.3). The PCR conditions were as follows: 94°C for 5 min; 94°C for 30 s, 52°C for 30 s, and 72°C for 120 s for 30 cycles; and 72°C for 10 min. The PCR products were detected *via* electrophoresis on 1.5% agarose gel pre-stained with ethidium bromide. The positive PCR amplicons were sequenced and compared with reported sequences from GenBank by Blast (www.ncbi.nlm.nih.gov/blast/).

### 2.5 String test

The string test was conducted as previously described (Yao et al., 2015). The KP isolates positive for hypermucoviscosity phenotypes were designated as HMKP. All isolates were cultured on blood agar plates (BIO-KONT, Wenzhou, China) incubated overnight at 37°C. An inoculating loop was used to touch the colonies gently and then lifted. A positive test was defined as a viscous string > 5 mm in length observed visually.

### 2.6 Capsular serotyping and detection of virulence-associated genes in carbapenem-resistant HMKP isolates

The primer sequences for capsular serotyping and virulence gene detection are listed in **Supplementary Data**. Capsular serotypes, including K1, K2, K5, K20, K54, and K57, were determined using the methods described previously (Turton et al., 2010). Three virulence-associated genes, including *rmpA*, *wcaG*, and *iutA*, were determined *via* PCR using the primers described previously for carbapenem-resistant HMKP isolates in **Supplementary Data** (Turton et al., 2010; Candan and Aksoz, 2015). NTUH-K2044 was used as the capsular serotype K1 positive control.

### 2.7 Conjugation and molecular cloning experiments

In total, 25 *bla*_KPC-2_, 4 *bla*_NDM-1_, 5 *bla*_NDM-5_, 1 *bla*_IMP-4_ and 4 *bla*_OXA-232_ of the CRKP were selected randomly for the conjugation experiment according to 10% of the single gene clinical strains. In the conjugation test, the donor bacteria included 39 clinical strains of CRKP insensitive to carbapenem. The conjugation experiment was carried out in Luria-Bertani (LB) medium (Oxoid) cultures, and the method has been described previously (Cai et al., 2008). Rifampin-resistant *E. coli* EC600 (LacZ^-^, Nal^R^, Rif^R^) was used as the recipient strain. Overnight cultures of the donor strain (200 μL) and recipient strain (100 μL) were mixed with 600 μL of fresh Mueller-Hinton broth and were incubated for 24 h at 35°C. Thereafter, the mixture was inoculated on MH agar plates containing rifampin (Sigma; 700 mg/L) and IPM (0.5 mg/L) for 24 h at 35°C. Colonies that grew on the selecting medium were isolated and identified by the MALDI-TOF MS (VITEK MS, bioMérieux). Plasmid DNAs were extracted from harvested and transformed *E. coli* using the alkaline lysis method and were sequenced by PCR using primers including those for *bla*_KPC_, *bla*_NDM_, *bla*_IMP_, and *bla*_OXA-48-like_, as described previously for CRKP (Poirel et al., 2011). The positive PCR amplicons were sequenced and compared with the reported sequences from GenBank by Blast (www.ncbi.nlm.nih.gov/blast/).

### 2.8 Virulence testing in the *Galleria mellonella* infection model

Selected strains containing the hypervirulent phenotype pattern of K2+iutA, K2+wcaG, K1+wcaG+iutA, K54+wcaG+iutA, K2+rmpA+iutA, K1+rmpA+wcaG+iutA, and K2+rmpA+wcaG+ iutA were randomly numbered *K. pneumoniae* 1-7 respectively. A *G. mellonella* model was used to assess the virulence of the seven *K. pneumoniae* isolates as previously described (McLaughlin et al., 2014; Chen et al., 2021). Overnight cultures of *K. pneumoniae* were diluted in sterile phosphate-buffered saline to obtain a concentration of 10^8^ CFU/ml. Wax moth larvae weighing 250 mg to 300 mg (Tianjin Huiyude Biotech Company, Tianjin, China) were injected with 10 μl bacterial suspension and incubated for 48 h at 35°C. *K. pneumoniae* strain NTUH-K2044 (GenBank accession NC_012731) was used as the positive control, and strain HS11286 (GenBank accession NC_016845) served as a low virulence control. The survival rate of *G. mellonella* was recorded at 18, 24, 42, and 48 h. Each isolate was tested in ten larvae, and all experiments were done in triplicate. Kaplan-Meier survival curves were plotted using GraphPad Prism.

### 2.9 Data analysis

Antimicrobial susceptibility data were analyzed using the WHONET 5.6 software (WHO, Geneva, Switzerland). Count data were expressed as rate (%). SPSS 23.0 software was used for data analysis and processing, and the χ2 test was applied for intergroup comparisons where *P* < 0.05 indicated a statistically significant difference.

## 3 Results

### 3.1 Antibiotic susceptibility test results

The resistance rates of CRKP isolates to CZA, tigecycline, and polymyxin B were all less than 30%. With the exception of amikacin (64.1%) and trimethoprim/sulfamethoxazole (56.7%), the resistance rates to all antimicrobial agents exceeded 75%. Resistance to CZA was not observed in isolates carrying single *bla*_KPC-2_ or *bla*_OXA-48-like_. However, resistance to CZA was at 100% in the *bla*_NDM_- and *bla*_IMP_-CRKP isolates that produced metallo-β-carbapenemases, with the difference being statistically significant (*P* < 0.05). Tigecycline and polymyxin B exhibited good antimicrobial activity in the overall isolate pool and in the various gene groups, with the resistance rates being lower than 5% and no statistically significant differences (*P* > 0.05) (**Table 1**).

**Table 1:** Antimicrobial resistance of clinical CRKP isolates (MICs, mg/L).

Antimicrobial agent	All isolates (n=417)					KPC-producers (n=251)				NDM-producers (n=86)				IMP-producers (n=9)				OXA-48-like producers (n=41)			
	MIC range	MIC_50_	MIC_90_	%R	%S	MIC_50_	MIC_90_	%R	%S	MIC_50_	MIC_90_	%R	%S	MIC_50_	MIC_90_	%R	%S	MIC_50_	MIC_90_	%R	%S
**PRL**	4->256	>256	>256	98.9	0.9	>256	>256	99.6	0.4	>256	>256	100	0	>256	>256	100	0	>256	>256	100	0
**TZP**	2- >256	>256	>256	98.1	1.4	>256	>256	98.2	1.6	>256	>256	98.8	0	>256	>256	100	0	>256	>256	100	0
**CIP**	0.06- >8	>8	>8	91.2	6	>8	>8	94.5	3.7	8	>8	82.3	12.8	8	>8	100	0	>8	>8	100	0
**LEV**	0.06- >16	>16	>16	89.9	8.3	>16	>16	94.1	4.8	4	>16	82.5	16.3	>16	>16	100	0	>16	>16	97.6	2.4
**CXM**	2- >64	>64	>64	100	0	>64	>64	100	0	>64	>64	100	0	>64	>64	100	0	>64	>64	100	0
**CRO**	0.12-64	>32	>32	100	0	>32	>32	100	0	>32	>32	100	0	>32	>32	100	0	>32	>32	100	0
**CAZ**	0.5->32	>32	>32	100	0	>32	>32	100	0	>32	>32	100	0	>32	>32	100	0	>32	>32	100	0
**FEP**	0.25->32	>32	>32	98.1	0.9	>32	>32	98.1	1	>32	>32	99.4	0	>32	>32	100	0	>32	>32	100	0
**ATM**	0.25->128	>128	>128	98.8	1	>128	>128	99.6	0.4	>128	>128	97.6	1.2	>128	>128	100	0	>128	>128	100	0
**CN**	1->128	128	>128	76	20.5	>128	>128	81.3	16.7	1	128	46.7	47.9	>128	>128	100	0	>128	>128	100	0
**AK**	1->128	8	>128	64.1	33.6	>128	>128	69.4	29.7	1	>128	23.4	70.6	>128	>128	100	0	>128	>128	100	0
**SXT**	0.25->32	32	>32	56.7	43.3	1	>32	46.9	53.1	>32	>32	56.3	43.7	>32	>32	88.9	11.1	>32	>32	100	0
**FOX**	1->64	>64	>64	93.5	3.3	>64	>64	94.4	3.2	>64	>64	97.4	1.2	>64	>64	100	0	>64	>64	93.3	4.9
**IPM**	0.12->16	16	>16	92.4	5.5	>16	>16	97.3	1.5	>16	>16	96.4	1.2	>16	>16	100	0	>16	>16	73.2	21.9
**MEM**	0.12->16	>16	>16	94.3	1.7	>16	>16	94.8	1.2	>16	>16	97.6	1.2	>16	>16	100	0	>16	>16	87.9	4.8
**CZA**	0.25->32	2	>32	26.4	73.4	0.5	4	0	100	>32	>32	100	0	>32	>32	100	0	0.5	4	0	100
**TGC**	0.12–8	0.5	2	1.2	93.8	0.5	2	0.8	94.1	0.5	2	1.2	95.3	0.5	2	0	88.9	0.5	2	2.4	92.8
**PB**	0.125->16	0.25	1	4.8	95.4	0.25	1	4.5	95.5	0.25	1	3.6	96.4	0.25	1	11.1	88.9	0.25	0.5	4.8	95.2

PRL, piperacillin; TZP, piperacillin/tazobactam; CIP, ciprofloxacin; LEV, levofloxacin; CXM, cefuroxime; CRO, ceftriaxone; CAZ, ceftazidime; FEP, cefepime; ATM, aztreonam; CN, gentamicin; AK, amikacin; SXT, trimethoprim/sulfamethoxazole; FOX, cefoxitin; IPM, imipenem; MEM, meropenem; CZA, ceftazidime/avibactam; TGC, tigecycline; PB, polymyxin B; CRKP, carbapenem-resistant K. pneumoniae. %R indicates the percentage of antimicrobial-resistant isolates; %S indicates the percentage of susceptible isolates.

### 3.2 Determination of carbapenemase types

**Figure 1:**
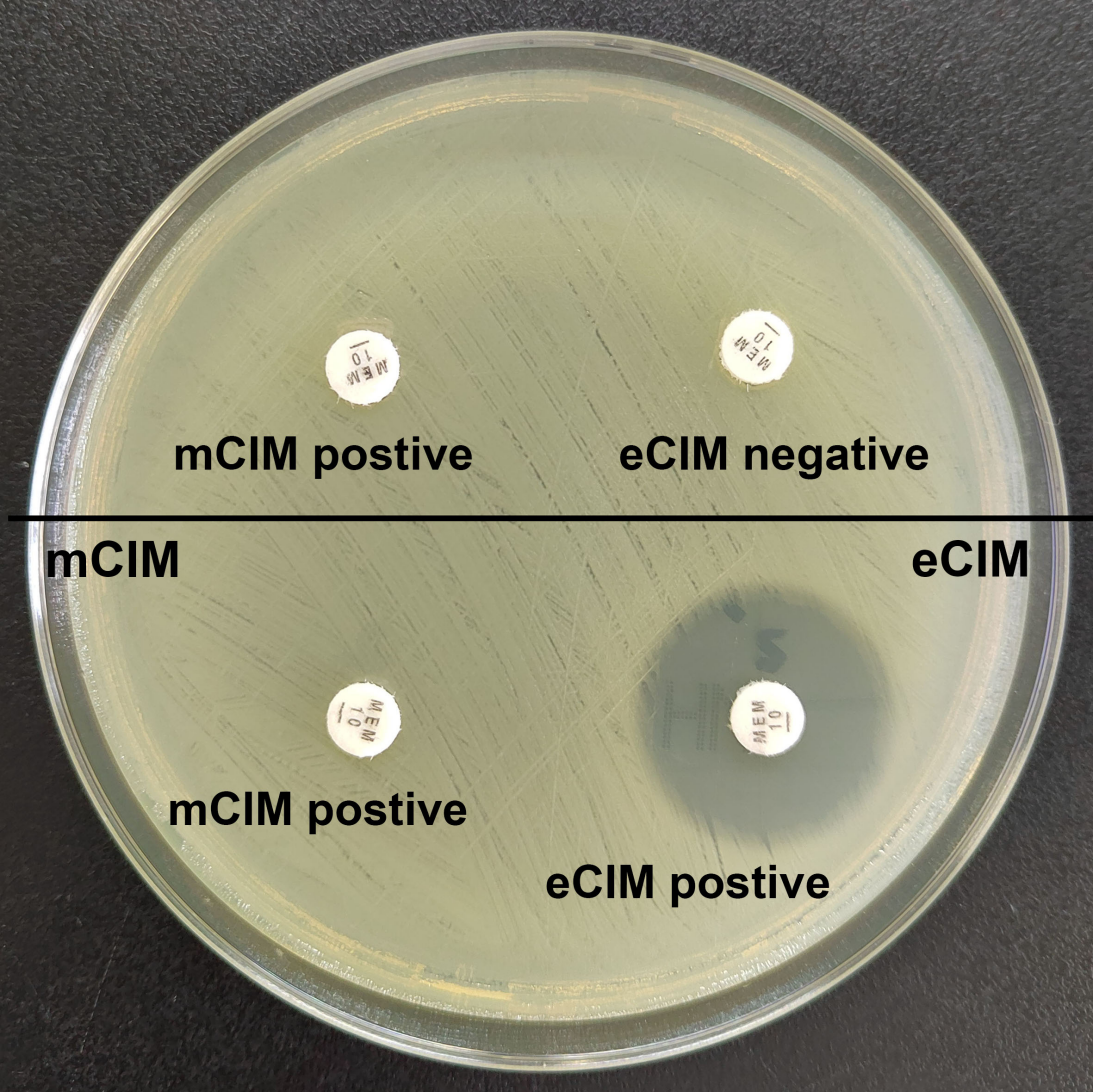
Results of mCIM/eCIM test of two CRKP isolates. The isolates in the upper half of the agar plate show positive mCIM and negative eCIM test results, indicating serine carbapenemase production. The isolates in the lower half of the agar plate show positive mCIM and positive eCIM test results, indicating MBL production.

Combined mCIM and eCIM testing of the 417 clinical CRKP isolates revealed that 402 isolates (96.4%) were mCIM-positive, and 110 (26.4%) were eCIM-positive (**Figure 1**). Furthermore, 292 serine carbapenemase-producing isolates (70.0%), 110 metallo-β-carbapenemase-producing isolates (26.4%), and 15 non-carbapenemase-producing isolates (3.6%) were observed (**Table 2**).

**Table 2:** Results of the combined mCIM-eCIM testing of clinical CRKP isolates.

	mCIM-positive (n)	mCIM-negative (n)
eCIM-positive	110	0
eCIM-negative	292	15

mCIM, modified carbapenem inactivation method; eCIM, EDTA-CIM method; CRKP, carbapenem-resistant K. pneumoniae.

### 3.3 PCR results for carbapenemase genes and transfer of carbapenem resistance and plasmid analysis

**Figure 2:**
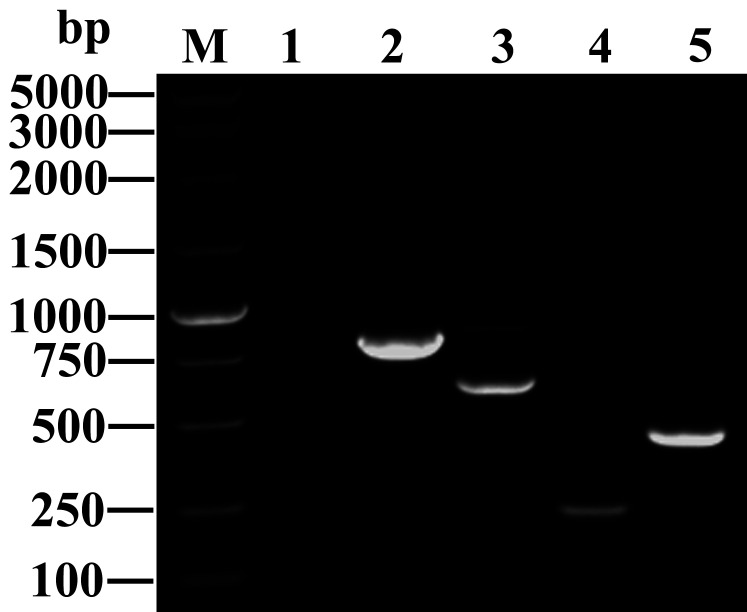
Amplified fragments of different carbapenemase genes. M: DNA marker (Takara Bio); Lane 1: blank control; Lanes 2 to 5: antibiotic resistance genes, including *bla*_KPC-2_ (798 bp), *bla*_NDM_ (621 bp), *bla*_IMP_ (232 bp), and *bla*_OXA-48_ (438 bp).

PCR revealed the presence of *bla*_KPC-2_, *bla*_NDM_, *bla*_IMP_, and *bla*_OXA-48-like_ in the 417 clinical CRKP isolates (**Figure 2**). The transfer of carbapenemase resistance from the 39 CRKP isolates to *E. coli* EC600 by conjugation was successful. All *E. coli* transconjugants exhibited significantly reduced carbapenem susceptibility with IMP and MEM MICs of 2 to 8 mg/L and an ETM MIC of 4 to 16 mg/L. They were also resistant or resistant to cefoxitin and cephalosporins. The corresponding carbapenemase resistance gene was detected in transformed *E. coli* by PCR and sequencing.

### 3.4 Carbapenemase gene detection rates and carriage patterns

The results showed that 96.4% (402/417) of the clinical CRKP isolates were positive for carbapenemase genes. The detection rate of *bla*_KPC-2_ (64.7%, 260/402) was significantly higher than that of *bla*_NDM_ (25.1%, 101/402; 12.4% for *bla*_NDM-1_ and 12.7% for *bla*_NDM-5_), *bla*_OXA-48-like_ (10.4%, 42/402; 10.2% for *bla*_OXA232_ and 0.2% for *bla*_OXA48_), and *bla*_IMP-4_ (4.2%, 17/402) (*P* < 0.05) (**Table 3**). Furthermore, 96.3% (387/402) of the isolates harbored a single carbapenemase gene, wherein 62.4% carried *bla*_KPC-2_, 21.4% carried *bla*_NDM_, 2.2% carried *bla*_IMP_, and 10.2% carried *bla*_OXA-48-like_), making it the dominant antibiotic resistance genotype carriage pattern (*P* < 0.05). The remaining 3.7% (15/402) of the isolates carried two or more carbapenemase genes, *bla*_KPC-2_ + *bla*_NDM-1_ (1.2%, 5/402) and *bla*_NDM-1_ + *bla*_IMP-4_ (1.2%, 5/402), as the main multiple antibiotic resistance genotype carriage patterns (**Table 3**).

**Table 3:** Detection rates and carriage patterns of carbapenem-resistant genotypes in clinical CRKP isolates.

Antibiotic resistance genotypes and carriage patterns	No. of isolates (n)	Percentage (%)
*bla*_KPC-2_	251	62.4
*bla*_NDM-1_	37	9.2
*bla*_NDM-5_	49	12.2
*bla*_IMP-4_	9	2.2
*bla*_OXA-232_	41	10.2
*bla*_KPC-2_ + *bla*_NDM-1_	5	1.2
*bla*_KPC-2_ + *bla*_NDM-5_	1	0.2
*bla*_NDM-1_ + *bla*_IMP-4_	5	1.2
*bla*_NDM-5_ + *bla*_OXA-48_	1	0.2
*bla*_KPC-2_ + *bla*_NDM-1_+*bla*_IMP-4_	3	0.7

GenBank accession, bla_KPC-2_/JF431928.1, bla_NDM-1_/JX506735.1, bla_NDM-5_/JAALBA010000102.1, bla_IMP-4_/JQ808503.1, bla_OXA-232_/MN652104.1, bla_OXA-48_/LC583818.1.

### 3.5 Carbapenem-resistant HMKP isolation

A total of 43 HMKP isolates were collected from specimens of blood cultures (n = 11; 25.6%), culture of sputum from the lower respiratory tract (n = 22; 51.2%), pus (n = 3; 7.0%), bile (n = 2; 4.6%), arteriovenous catheter (n = 2; 4.6%), and sterile fluids, including articular fluid, hydrothorax, and ascitic fluid (n = 3; 7.0%).

### 3.6 Capsular serotyping and prevalence of virulence-associated genes in carbapenem-resistant HMKP isolates

**Figure 3:**
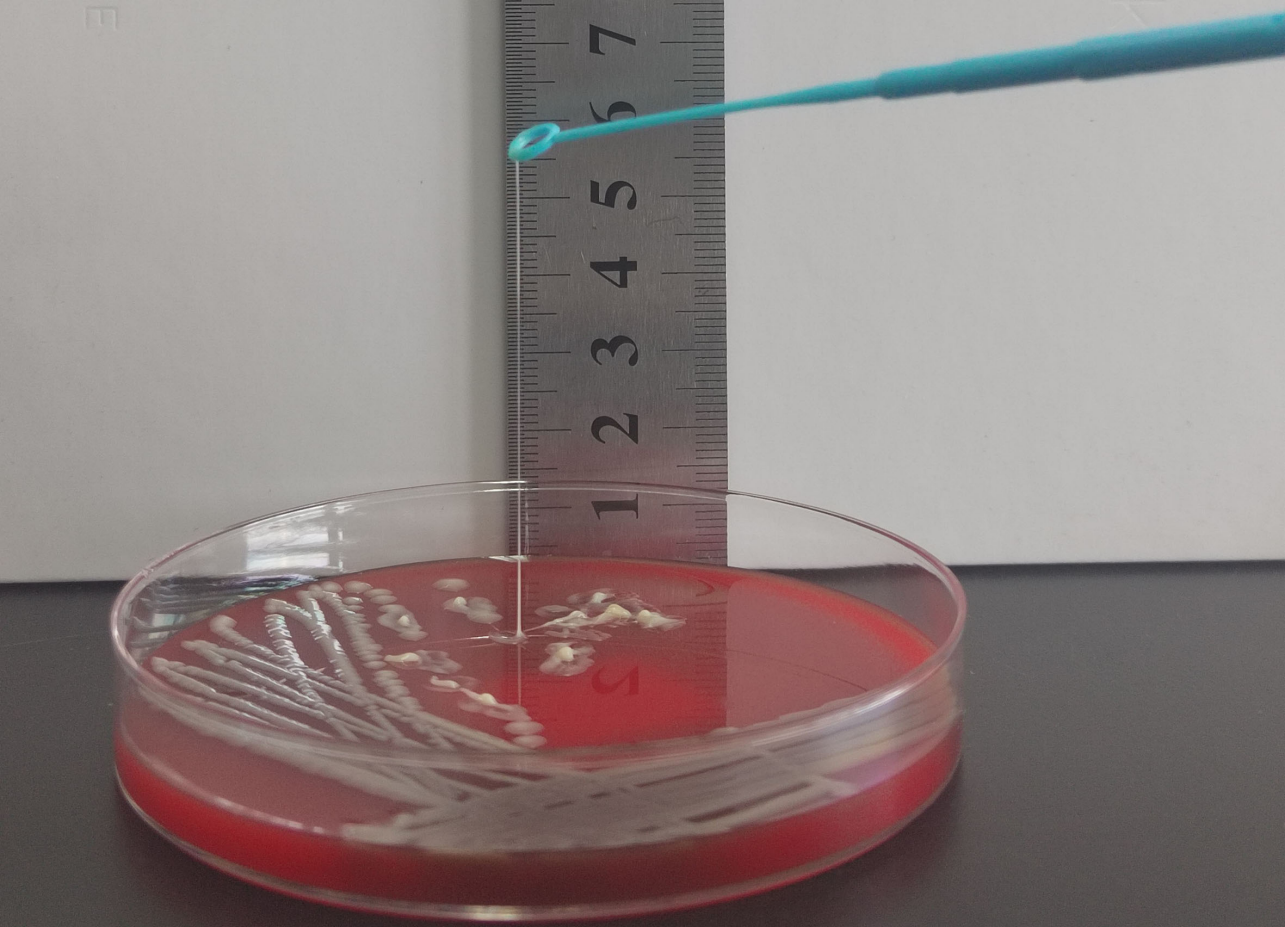
String test for identification of the HMV phenotype. A positive string test is defined as the formation of viscous strings of > 5 mm in length on an agar plate.

Among the 417 CRKP isolates, 43 (10.3%) were carbapenem-resistant HMKP isolates (**Figure 3**), including those belonging to capsular serotype K2 (46.5%, 20/43), capsular serotype K1 (27.9%, 12/43), and capsular serotype K54 (25.6%, 11/43). The virulence-associated genes among the 43 isolates included *wcaG* (65.1%, 28/43), *rmpA* (39.5%, 17/43), and *iutA* (95.3%, 41/43) (**Table 4**).

**Table 4:** Virulence genotypes and carrying mode in 43 carbapenem-resistant HMKP isolates.

Virulence genotypes and carriage mode	No. of isolates (n)	Percentage (%)
K2+*iutA*	8	18.6
K2+*wcaG*	2	4.7
K1+*wcaG*+*iutA*	5	11.6
K54+*wcaG*+*iutA*	11	25.6
K2+*rmpA*+*iutA*	7	16.3
K1+*rmpA*+*wcaG*+*iutA*	7	16.3
K2+*rmpA*+*wcaG*+*iutA*	3	7.0

### 3.7 Virulence analysis using the *G. mellonella* infection model

**Figure 4:**
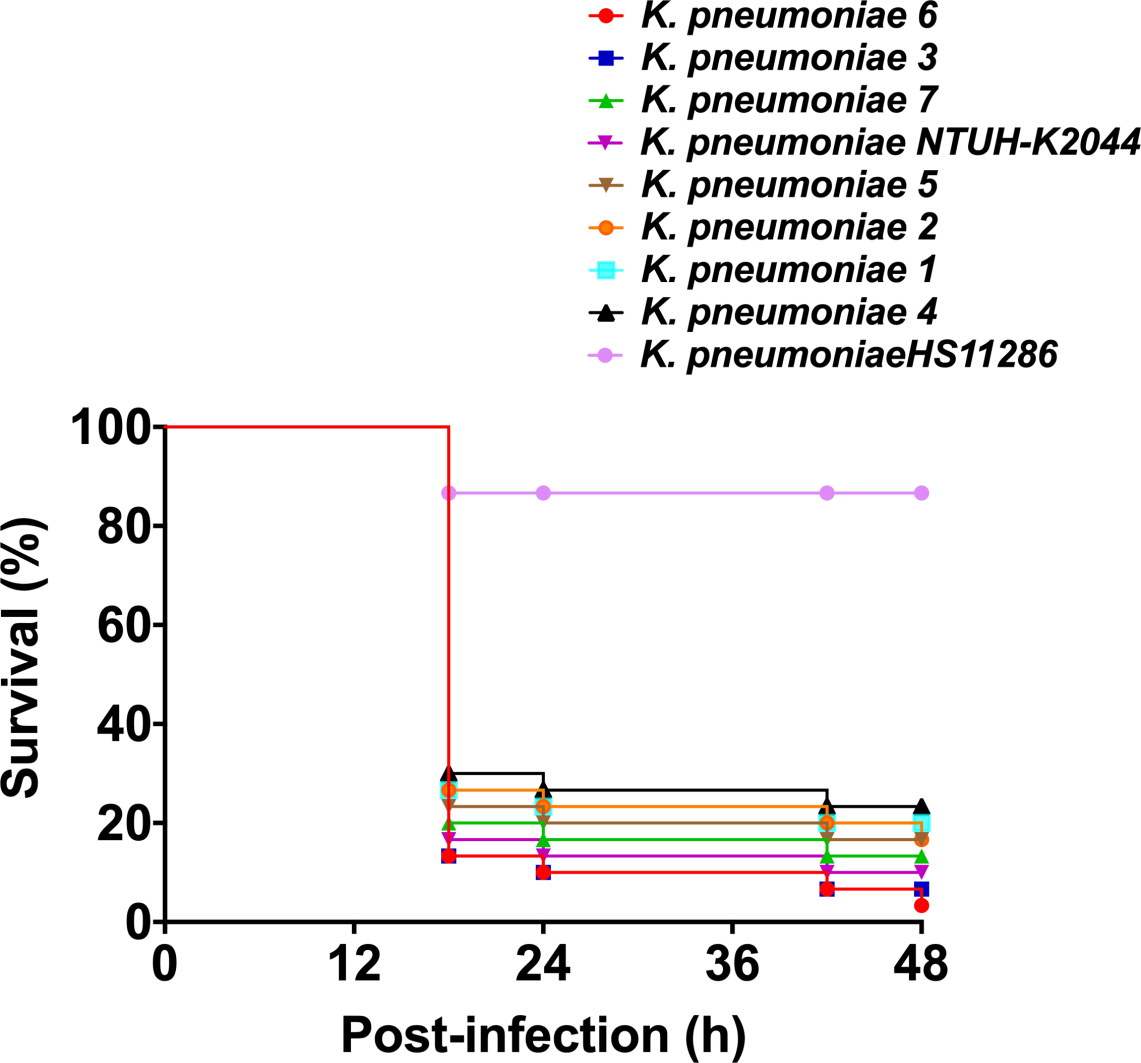
Virulence of *K. pneumoniae* isolates in the *G. mellonella* infection model.

The hypervirulent phenotype in the seven CR-hvKP was observed in the *G. mellonella* infection model. Survival rates of the larvae injected with KP 1–7 (an inoculum of 1 × 10^6^ CFU) at 48 h after infection were significantly lower than that of negative control isolate *K. pneumoniae* HS11286 (20.0%, 16.7%, 6.7%, 23.3%, 16.7%, 3.3%, and 13.3%, respectively, vs. 86.7%; **Figure 4**).

### 3.8 Risk factors associated with clinical characteristics caused by carbapenem-resistant HMKP isolates

Recent antibiotic therapy (within 1 month prior to mechanical ventilation) and previous hospitalization of 5 days or more (within invasive procedure) were significant risk factors for carbapenem-resistant HMKP isolates among the clinical characteristics (**Table 5**).

**Table 5:** Risk factors associated with clinical characteristics caused by carbapenem-resistant HMKP isolates.

Risk Factors	*K. pneumoniae* Isolates (n = 417)				*χ*2	*P*
	HMV-CR*K. pneumoniae*		Non HMV-CR*K. pneumoniae*			
	(n = 43)		(n = 374)			
	n	%	n	%		
Age						
<70 (n = 69)	10	23.3	59	15.8	1.56	0.211
≥70 (n = 348)	33	76.7	315	84.2		
Previous hospitalization of 5 days or more						
Yes (n = 334)	25	58.1	309	82.6	14.50	0.000*
No (n = 83)	18	41.9	65	17.4		
Duration of mechanical ventilation						
Yes (n = 244)	19	44.2	225	60.2	4.05	0.044*
No (n = 173)	24	55.8	149	39.8		
Associated comorbidities						
Yes (n = 373)	40	93.0	333	89.0	0.65	0.420
No (n = 44)	3	7.0	41	11.0		
Invasive procedures						
Yes (n = 268)	21	48.8	247	66.0	4.97	0.026*
No (n = 149)	22	51.2	127	34.0		
Recent antibiotics intake						
Yes (n = 338)	26	60.5	312	83.4	13.24	0.000*
No (n = 79)	17	39.5	62	16.6

*Statistically significant at p < 0.05.

## 4 Discussion

Currently, CRKP and carbapenem-resistant HMKP isolates, associated with considerable morbidity and mortality, are a major threat to public health worldwide (Wyres et al., 2020). Our study has described the prevalence of CRKP and carbapenem-resistant HMKP isolates from a tertiary hospital in China and characterized the drug resistance, virulence factors, and risk factors of these isolates. Previous studies reported that carbapenemase production was the primary mechanism underlying CRKP drug resistance (Gandra and Burnham, 2020; Karaiskos et al., 2021). CZA, a combination of ceftazidime (cephalosporin) and avibactam (novel synthetic β-lactamase inhibitor), was approved in the Chinese market in May 2019 and serves as an effective option for the treatment of KPC- or OXA-48–producing KP infection (Mehmood et al., 2020; Mataraci et al., 2020). Our results showed that the rates of resistance to tigecycline and polymyxin B in CRKP were 1.2% and 4.8%, respectively, which demonstrates the emergence of drug-resistant isolates at our hospital following the prolonged use of antimicrobials. The rate of resistance to CZA in our studied isolates (26.4%) was lower than that reported in a previous study, which may be attributed to the presence of carbapenem-resistant Enterobacterales isolates producing high levels of NDM (Han et al., 2020). These patterns differ considerably from those observed in Europe. Grundmann et al. (2017) reported that the prevalence rate of OXA-48-producing Enterobacterales was 38% (333/927), which was second to the prevalence rate of KPC-producing Enterobacterales (42%, 393/927), but higher than that of NDM-producing Enterobacterales (12%, 113/927). In the present study, we also observed the absence of CZA-resistant isolates among those that produced KPC-2 or OXA-48 only. Our findings indicate that the carriage of a single gene was the dominant antibiotic genotype carriage pattern in the clinical CRKP isolates from our hospital, which was consistent with the results reported by two previous studies (Bi et al., 2019; Han et al., 2020). In total, 15 of the 417 clinical isolates did not produce carbapenemases; their resistance to carbapenems may be largely related to the combined effects of the absence of membrane porins and other mechanisms (Bulman et al., 2021).

The detection of metallo-β-lactamase-producing carbapenem-resistant Enterobacterales (CRE) strains has limited the clinical applications of CZA, but also promoted the development of novel therapies or combination therapies against drug-resistant CRE strains. For instance, Biagi et al. (2019) reported that aztreonam combined with MEM/vaborbactam or CZA exhibited good antimicrobial activity against NDM-producing CRE strains and non-OXA-48-expressing Enterobacterales. Another report demonstrated that the combination of colistin and amikacin exhibited sustained antimicrobial activity against *E. coli* co-producing NDM-5 and MCR-1 and may serve as an alternative therapeutic option for the treatment of lethal infections (Zhou et al., 2017).

Furthermore, our results showed that KPC-2, NDM, and OXA-48-like were the most common carbapenemases in the CRKP strains isolated from our hospital, with *bla*_KPC-2_ being the main carbapenemase gene. All CRKP isolates were highly resistant to cephalosporins, carbapenems, and fluoroquinolones, but showed higher susceptibility to polymyxin B and tetracycline. CZA exhibited good antimicrobial effects against clinical CRKP isolates that carried a single *bla*_KPC-2_ or *bla*_OXA-48-like_ gene, among which CZA-resistant isolates were absent. The susceptibility rate demonstrated by the present study exceeds the CZA susceptibility of 92.6% previously reported (Kazmierczak et al., 2018). Given the high drug resistance, mortality rate, and anti-infective treatment costs, as well as the difficulty in eradicating CRKP strains from the host, enhanced monitoring of carbapenemase-resistant strains and their prevalence by clinicians is required for the adoption of practical and effective measures to prevent the spread of highly resistant isolates.

Other previous reports suggested that acquiring high virulence and carbapenem resistance in KP was increasingly becoming a concern in hospitals in China (Zhan et al., 2017; Li et al., 2020a). With the increasing aging of the Chinese population, this study showed that such infections are one of the factors that seriously affect health. Hypermucoviscosity and hypervirulence are two different KP phenotypes (Catalán-Nájera et al., 2017). The string test is widely used to identify hypervirulent KP(hvKP). A few classical KP isolates tested positive in the string test, despite being categorized as low virulence isolates. The sensitivity, specificity, and accuracy of the sting test are 95.56, 91.38, and 93.20%, respectively (Li et al., 2020b). Taken together, this study indicated that the string test is still a reliable although imperfect method. A report from Hangzhou, China showed that among 42 carbapenem-resistant HMKP isolates, 16 (38.1%) belonged to K1, 5 (11.9%) belonged to K2, and 11 (26.2%) were non-typable (Chen et al., 2018). Another study from Wenzhou, China found that only one carbapenem-resistant HMKP isolate belonged to K2 (4.5%, 1/22), and that 33.3% (7/21) of the isolates belonged to K20 (Zhan et al., 2017). Similarly, in the present study, K2 was the main capsular serotype and accounted for 46.5% (20/43) of the isolates, followed by K1 (27.9%, 12/43). Moreover, we identified that 25.6% (11/43) of the carbapenem-resistant HMKP isolates belonged to K54, suggesting that K54 may be an important capsular serotype associated with carbapenem-resistant HMKP. A report from China described that *rmpA* was present in nearly all HMKP isolates (97.6%, 41/42) (Chen et al., 2018). Herein, 39.5% (17/43) of the carbapenem-resistant HMKP isolates harbored *rmpA*.

Previous studies have shown that two capsule regulator genes, *rmpA* and *rmpA2*, are hvKP-specific virulence factors in terms of the abscess formation of HMKP isolates on the virulence plasmid (Li et al., 2020b). For instance, *wcaG* and *magA* are associated with capsule biosynthesis (Zhang et al., 2019). Siderophores (SPs) are also essential virulence genes, which can be released from HvKP strains invading the host body. SPs can acquire iron from the binding protein in the host body and can then bind to the receptor to re-enter cells. Four SPs secreted by hvKP, namely salmochelin, enterobactin, yersiniabactin, and aerobactin, have been reported previously (Chen et al., 2018; Han et al., 2022). The function of aerobactin gene *iutA* is well-correlated with the *ex vivo* growth/survival of hvKP in humans (Russo et al., 2015). Hence, SP-related encoding genes, especially *iutA*, are key markers in the identification of hvKP (Russo et al., 2015; Han et al., 2022). Many virulence-based experiments may be required for isolates from hvKP *in vitro* and *in vivo*, such as in mice models, greater wax moth (*G. mellonella*) models, and neutrophil phagocytosis assays. Mouse infection models are widely used to evaluate the virulence of pathogens but have disadvantages, including high expenses and requirement for an animal biosafety level-2 facility environment (Zhang et al., 2019). *G. mellonella*, a high-throughput animal model developed over the last 20 years, can also be used to determine bacterial pathogenicity (Zhang et al., 2019; Chen et al., 2021). *G. mellonella* larvae are easily obtained, inexpensive, and can be maintained in a wide temperature range (Zhang et al., 2019). Hence, *G. mellonella*, is being increasingly used as a model for evaluating the pathogenicity of various microorganisms. This model serves as an alternative system that provides comparable data and is more cost-effective than mammalian testing, ethically acceptable, and highly beneficial. The study adopted the *G. mellonella* infection model, which can be used to determine bacterial pathogenicity in view of these advantages. Here, we present data indicating that the *G. mellonella* model is an easily manageable system for comparing the virulence of different *K. pneumoniae* isolates.

This study evaluated the risk factors associated with clinical characteristics caused by carbapenem-resistant HMKP isolates. Most of the carbapenem-resistant HMKP isolates were associated with significant risk factors such as prior antibiotic therapy, previous hospitalization for 5 days or more, invasive procedures, and mechanical ventilation. Other studies have reported that prolonged hospitalization, invasive procedures (e.g., central or urinary catheter insertion, tracheostomy, and invasive abdominal procedures), serious underlying diseases (e.g., diabetes mellitus, hemiplegia, and solid tumors), and prior carbapenem exposure, were independent predictors for infection development in patients with carbapenem-resistant HMKP isolates (Borer et al., 2012; Chen et al., 2018; Han et al., 2022).

Our study had several limitations. First, multilocus sequence typing and pulsed-field gel electrophoresis clustering were not performed using the CRKP isolates; therefore, the link among the strains could not be divided into different groups. Second, the number of carbapenemase, capsule, and virulence-related genes tested was limited. Third, this is a retrospective study that was conducted in a single hospital.

In conclusion, the resistance rate of CRKP to β-lactam antibiotics was higher than 94%, except for CZA, and it is increasing annually. KPC-2, NDM, and OXA-48-like were the most prevalent carbapenemases among CRKP clinical isolates in China. A small number of the strains carried two or more carbapenemase genotypes. The newly available β-lactam combination agent, CZA, demonstrated good *in vitro* activity against KPC-2 or OXA-48-like-producing isolates and showed promise in treating CRKP infections. K1 and K2 were the main capsular serotypes of the HMKP strains. Considering that CRKP is highly resistant to commonly used antibiotics, antibiotics for clinical treatment of CRKP infections should be selected following drug sensitivity tests, and hospital administrators should provide training and assessment to clinicians on the rational use of antibiotics to reduce the infection rate of multidrug-resistant bacteria in the future. Our results provide evidence for the investigation and prevention of CRKP infections worldwide.

## Data availability statement

The raw data supporting the conclusions of this article will be made available by the authors, without undue reservation.

## Ethics statement

The studies involving human participants were reviewed and approved by the Ethics Committee (IRB) of the Second Affiliated Hospital of Zhejiang Chinese Medical University. Written informed consent for participation was not required for this study in accordance with the national legislation and the institutional requirements. The animal study was reviewed and approved by the Ethics Committee (IRB) of the Second Affiliated Hospital of Zhejiang Chinese Medical University. No potentially identifiable human images or data is presented in this study.

## Author contributions

XT and DJ conceived and designed the experiments. QW, MC, GM, and JF performed the experiments. QO, LZ, and XC analyzed the data. QW and MC wrote the manuscript. All authors read and approved the final manuscript. All authors contributed toward data analysis, drafting and revising the manuscript, and agreed to be accountable for all aspects of the work. All authors contributed to the article and approved the submitted version.
