# Supplementary material for: Carbapenem-resistant hypermucoviscous Klebsiella pneumoniae clinical isolates from a tertiary hospital in China: Antimicrobial susceptibility, resistance phenotype, epidemiological characteristics, microbial virulence, and risk factors
Source: Front Cell Infect Microbiol. 2022 Dec 21;12:1083009. doi: 10.3389/fcimb.2022.1083009 (PMC9811262; doi:10.3389/fcimb.2022.1083009)
Supplement: Supplementary file 1 [file Table_1.docx]

**Table S1** Primers used in polymerase chain reaction and their sequences; the sequences of primers for resistance genes, capsular serotyping and virulence-associated genes

| Primer | Primer sequence (5’-3’) | T_m_* (℃) | Product size (bp) | Refrences |  |
| --- | --- | --- | --- | --- | --- |
| *bla*_KPC_ | F: CGTCTAGTTCTGCTGTCTTG | 52.0 | 798 | Poirel et al., 2011 |  |
|  | R: CTTGTCATCCTTGTTAGGCG |  |  |  |  |
| *bla*_NDM_ | F: GGTTTGGCGATCTGGTTTTC | 52.0 | 621 | Poirel et al., 2011 |  |
|  | R: CGGAATGGCTCATCACGATC |  |  |  |  |
| *bla*_IMP_ | F: GGAATAGAGTGGCTTAAYTCTC | 52.0 | 232 | Poirel et al., 2011 |  |
|  | R: GGTTTAAYAAAACAACCACC |  |  |  |  |
| *bla*_OXA-48-like_ | F: GCGTGGTTAAGGATGAACAC | 52.0 | 438 | Poirel et al., 2011 |  |
|  | R: CATCAAGTTCAACCCAACCG |  |  |  |  |
| *rmpA* | F:ACTGGGCTACCTCTGCTTCA | 64.0 | 516 | Turton et al., 2010 |  |
|  | R: CTTGCATGAGCCATCTTTCA |  |  |  |  |
| *wcaG* | F: GGTTGGKTCAGCAATCGTA | 65.1 | 169 | Turton et al., 2010 |  |
|  | R:ACTATTCCGCCAACTTTTGC |  |  |  |  |
| *iutA* | F:GGCTGGACATCATGGGAACTGG | 66.0 | 300 | Candan and Aksoz, 2015 |  |
|  | R:CGTCGGGAACGGGTAGAATCG |  |  |  |  |
| Capsular type K1 | F: GGTGCTCTTTACATCATTGC | 59.7 | 1283 | Turton et al., 2010 |  |
|  | R: GCAATGGCCATTTGCGTTAG |  |  |  |  |
| Capsular type K2 | F: GACCCGATATTCATACTTGACAGAG | 63.5 | 641 | Turton et al., 2010 |  |
|  | R:CCTGAAGTAAAATCGTAAATAGATGGC |  |  |  |  |
| Capsular type K5 | F: TGGTAGTGATGCTCGCGA | 64.4 | 280 | Turton et al., 2010 |  |
|  | R: CCTGAACCCACCCCAATC |  |  |  |  |
| Capsular type K20 | F: CGGTGCTACAGTGCATCATT | 63.7 | 741 | Turton et al., 2010 |  |
|  | R: GTTATACGATGCTCAGTCGC |  |  |  |  |
| Capsular type K54 | F: CATTAGCTCAGTGGTTGGCT | 61.8 | 881 | Turton et al., 2010 |  |
|  | R: GCTTGACAAACACCATAGCAG |  |  |  |  |
| Capsular type K57 | F: CTCAGGGCTAGAAGTGTCAT | 58.5 | 1037 | Turton et al., 2010 |  |
|  | R: CACTAACCCAGAAAGTCGAG |  |  |  |  |

F: forward primer; R: reverse primer
